# Youth and Adult Visitation and Physical Activity Intensity at Rural and Urban Parks

**DOI:** 10.3390/ijerph15081760

**Published:** 2018-08-16

**Authors:** James N. Roemmich, LuAnn Johnson, Grace Oberg, Joley E. Beeler, Kelsey E. Ufholz

**Affiliations:** Grand Forks Human Nutrition Research Center, Agricultural Research Service, U.S. Department of Agriculture, Grand Forks, ND 58203-9034, USA; james.roemmich@ars.usda.gov (J.N.R.); luann.johnson@ars.usda.gov (L.J.); gpuppe@grandforksgov.com (G.O.); joley.beeler@my.und.edu (J.E.B.)

**Keywords:** built environment, exercise, children, adolescents, adults, physical activity, rural, urban

## Abstract

Less physical activity among rural residents may contribute to rural-urban health disparities. Parks can be ideal community resources for promoting physical activity. This study compared park visitation and activity intensity at 15 urban and 15 rural parks matched for acreage and amenities. Parks were observed in the morning, afternoon, and evening on 4 days to determine number of visitors, activity intensity, and amenity use. A total of 5486 visitors were observed with no differences in percentages of males (55.5% vs. 53.9%) and females (44.5% vs. 46.1%) or percentages of weekday (82.4% vs. 81.9%) and weekend (17.6% vs. 18.1%) visitors. The probability of visitors sitting was greater and in moderate intensity activity lower at rural parks. A greater proportion of children (25.0% vs. 14.5%) in rural parks, and teens in urban parks (8.0% vs. 69.6%), were observed on sport fields. A greater proportion of adults in urban areas (12.5% vs. 46.0%) were observed spectating sports. Greater proportions of rural children (10.9% vs. 3.5%), teens (34.1% vs. 12.4%), and adults (38.9% vs. 10.1%) were observed using shelters. Thus, when similar amenities are available, rural and urban parks are used differently, especially by youth. The urban park study results cannot be wholly applied to rural parks.

## 1. Introduction

Participation in physical activity provides mental and physical health benefits and reduces the risk of many chronic diseases [1,2]. However, a disparity exists in the physical activity levels of rural and urban residents. Rural youth and adults are less likely to engage in leisure time physical activity compared to urban and suburban adults, even when controlling for race, education, and income [3,4] such that they may not receive all of its health benefits. As such, rural residency increases the risk of overweight and obesity in youth [5] and adults [6]. These health disparities between rural and urban residents may be due to factors that promote differential physical activity in rural and urban environments. Considering that 21% of the U.S. population lives in a rural environment, it is important to determine the factors that may promote sedentary behavior or physical activity of rural residents.

A growing body of evidence demonstrates that design factors of the built environment can promote either sedentary [7,8] or, in the case of parks, physically active behaviors [9,10]. For parks to promote activity, people must make the choice to visit a park and to be active when at the park. Health behavior choices, including physical activity, of residents in rural communities are dependent on access to physical activity-related amenities and programming within rural communities. Recent studies have examined differences in urban and rural residents’ physical activity using GPS [11], surveys [12], and focus groups [11]. These studies have demonstrated that, in addition to demographic factors such as age, gender, and socioeconomic status [13,14,15], the geographical setting in which a person lives can influence the manner in which the built environment promotes physical activity and health behaviors [11]. This choice is likely dependent on the park’s condition, amenities, design characteristics, and proximate access to a park [9,10,16]. Thus, using a singular approach to promote physical activity in both urban and rural settings is likely to be ineffective, and may offer little benefit to many residents’ health.

Park use of those living in rural and urban communities might be anticipated to differ based on potential cultural differences. Certainly, there is wide diversity in the health behaviors among individuals living in both rural and urban communities; and between communities as a whole regardless of setting or population. Still, some cultural factors that may act as barriers to physical activity in rural communities include distances to parks and other physical activity outlets, and no or smaller social networks to provide support for wellness and park-based physical activity [17,18]. The current investigation compares visitation, amenity use, and physical activity intensity between urban and rural parks matched for acreage and available amenities.

## 2. Methods

### 2.1. Setting

During the summer of 2014, physical activity type and intensity of park users was investigated at 15 urban parks in Grand Forks, ND, and East Grand Forks, MN, and 15 parks in rural communities in Grand Forks, Nelson, Pembina, Steele, Trail, and Walsh counties of ND. The cities of Grand Forks and East Grand Forks have populations of 54,932 and 8602 people, respectively and reside in county-levels coded as 3 of 9 (“counties in metro areas of fewer than 250,000 population”) according to the 2013 USDA Economic Research Service Rural–Urban Continuum Code [19]. The rural parks resided in communities situated a median 39.7 miles (range 13.9 to 65.1 miles) from Grand Forks/East Grand Forks in counties with a median 2013 USDA Economic Research Service Rural–Urban Continuum Code of 8 of 9 (“completely rural or less than 2500 urban population, adjacent to a metro area”). Median population density of the rural park communities was 9.4 (range 2.8 to 47.0) persons per square mile. Parks were included in the study by first assessing the attributes of each park in Grand Forks/East Grand Forks metro area and the rural North Dakota parks within 70 miles of metro area. The acreage and each amenity of each park were then detailed in a spreadsheet with the name of each rural park at the top of a column, and each amenity in a separate row below the park name. A given row represented a specific amenity type and was color-coded so that if the amenity was present at a park, then the cell in that row was filled with the color assigned to that amenity. If the park did not have an amenity, then the cell was empty. The same process was completed for the urban parks. Then, because there were more urban than rural parks, the urban parks were matched as best as possible with the rural parks, based on acreage and available amenities (Table 1). Eight urban parks, most with only a playground and greenspace, were not included in the analysis because there was no rural park match. The study was conducted in accordance with the Declaration of Helsinki, and the protocol was approved by the University of North Dakota Institutional Review Board (IRB-201406-489).

### 2.2. Visitation

Observers were trained in the use of the System of Observing Play and Recreation in Communities (SOPARC), which allows for measurement of the number of people of specified demographics (e.g., age and gender) engaged in physical activities of sedentary, moderate or vigorous intensity. Training involved the use of a standardized SOPARC DVD and training materials [20] followed by several days of field training. Training continued until interobserver reliability reached at least 80% agreement with the trainer (JNR) for demographics, number of people observed per scan, and activity intensity. Construct validity of SOPARC activity intensity has been established using heart rate as the criterion [21].

### 2.3. Data Collection

Park areas were sectioned into component parts to ease scoring when many visitors were present, and to allow for analysis by amenity. Systematic observations of the target areas took place 3 times per day (periods starting at 10:30, 14:30, and 17:30) for 3 weekdays and 1 weekend day, for a total of 12 observations/park. Three trained observers completed the park observations. Observation periods were completed by a single observer and all 3 daily sessions for a park were completed on the same day, unless rescheduling was necessary due to weather (e.g., raining, real temperature of less than 15 C). All parks were observed at the same times of the day, but due to the number of parks observed (*n* = 30) and the great travel distances between the parks, the observations for all parks could not be completed on the same days. An observation schedule of 4 days/week with 4 observation periods/day reliably estimates park use by gender, age, and physical activity intensity as measured when observing hourly for 14 h/day on 7 consecutive days [22]. The current study completed 3 observations per day. Previous research has used 3 observations periods/day with comparable results to those studies that used 4 observations periods/day [23]. Observer(s) performed rapid visual scans to determine the number of children and adults and their gender, age category (young child 0–5 yrs, child 6–12 yrs, teen 13–18 yrs, and adult 19+ yrs), activity intensity (sitting, standing, walking/moderate, or vigorous), and location of activity (e.g., specific park amenities: e.g., shelters, playgrounds, sports fields, etc.). Intensity was scored at the moment of observation and not of the general activity. Activity level classifications were converted to metabolic equivalent of task (MET) intensities (sitting = 1.25 METs; standing = 1.5 METs; moderate = 3.0 METs; vigorous = 6.0 METs) based on previously described values [20,24,25]. Observers did not interact with the patrons, except to acknowledge and return perfunctory greetings. Park visitors were not informed of the research as this might elicit a change in behavior.

### 2.4. Programming

Park programming was assessed using park programming brochures, Facebook, websites, city calendars, and by contacting Park Boards and managerial staff. The number of days that each program was available during the summer months were summed (i.e., youth softball available 2 times per week for 6 weeks received a score of 12). Programs were categorized by age group (0–12 yrs, 13–18 yrs, 19+ yrs) of the intended participants. The total number of program days for each park for each age group was calculated by summing the program days of all events held in that park. Multiple events held on one day were counted as multiple days of programming. Programming that could not be consistently and accurately captured across both the urban and rural parks, such as single day events (e.g., fun runs, large picnic gatherings, community celebration events), were not included in the programming totals.

### 2.5. Park Assessments

The amenities present and the quality of the parks were assessed using the Public Open Space Tool (POST) [26] and the Environmental Assessment of Public Recreation Spaces (EAPRS) [27]. For both assessments, trained observers visually inspected, in-person, each park for each item. POST assesses park facilities used for both active recreation and sedentary activities. However, the POST score is based on 10 park attributes (e.g., water feature(s), shade trees along paths, walking paths, sport courts, playgrounds, lighting) associated with park-based physical activity. The attributes are individually and unequally weighted such that a maximum POST score is 100 points [26]. EAPRS provides a comprehensive direct observation assessment of the physical environments of parks and playgrounds, with an emphasis of evaluating physical elements and qualities with respect to their functionality. EAPRS provides a summary or total score. EAPRS has a minimum score of 11 and max score of 2595. The EAPRS “physical activity quality” subscore, which assesses the quality and function of amenities that promote physical activity (e.g., sports fields, courts, playsets, paths), the “playground” subscore, which provides a measure of the amount and quality of equipment on the playground, and the “access” and “sidewalk safety” subscores, were also utilized in the present study.

### 2.6. Neighborhood Assessments

Walkability, or the ease and safety of walking or biking to each park, was assessed using the Irvine–Minnesota Inventory (IMI; [28,29]). The IMI includes 162 items, organized into four domains: accessibility (62 items), pleasurability (56 items), perceived safety from traffic (31 items), and perceived safety from crime (15 items). Trained observers used the IMI to assess 5 intersections and 2 segments per intersection within ½ mile of each park. The intersections were chosen to provide a reflection of the built design aspects of the neighborhoods, including roadways with low and high traffic, schools, businesses, and residential areas. The average intersection walk score from the IMI was used to index walkability of the neighborhood around each park. Neighborhood population was based on 2010 census block group data within which the park was located. If a park spanned census block groups, then the block group that the majority of the park area resided in was used as data. Many of the rural communities were composed of a single census block group.

## 3. Analysis

Fifteen urban parks were matched to 15 rural parks based on acreage and number of amenities. Paired *t*-tests were used to compare park characteristics between the matched pairs of rural and urban parks. The overall MET intensity of park visits and the visits per amenity were analyzed using analysis of covariance with setting (rural or urban) as the fixed effect, park pair as a random effect, and neighborhood population as the covariate. Due to its highly skewed distribution, visits per amenity were log-transformed prior to analysis. Visitation patterns between rural and urban parks were compared using a generalized linear mixed model in which setting (rural or urban) was the fixed effect and park pair was the random effect. Percent of visitors by gender and day of week were modeled using the binomial distribution, while the multinomial distribution was used to test for differences by time of day, age category, and activity intensity. The percentage of visitors at specific park amenities was analyzed using the binomial distribution in a generalized linear model in which setting, age group, and their interaction were fixed effects. Data were too sparse to include park pair as a random effect in these models. Additionally, because the unit of analysis was parks (*N* = 30), the sample was insufficient for multivariate analyses of park characteristics. However, given the limited number of parks available, an increased sample size was not possible. The Glimmix procedure in SAS V9.4 (SAS Institute, Inc., Cary, NC, USA) was used for all of the above analyses.

## 4. Results

As expected, due to the matched-pair study design, as shown in Table 2, the parks did not differ for acreage (*p* ≥ 0.72), POST score (*p* ≥ 0.52), total EAPRS score (*p* ≥ 0.17), or EAPRS playset (*p* ≥ 0.24) subscore. The rural parks scored lower for EAPRS physical active quality subscore (*p* < 0.006), access (*p* < 0.001), and sidewalk safety (*p* < 0.001). Programming did not differ between the rural and urban parks (*p* > 0.95). The neighborhoods around the urban parks had a greater (*p* < 0.001) population and greater (*p* < 0.03) neighborhood walkability score.

Visitation patterns of the parks are shown in Table 3. There were no differences between rural and urban parks for the percentage of male and female visitors (*p* > 0.77), and weekday versus weekend (*p* > 0.96) visitation. A greater (*p* < 0.001) percentage of the daily visitors were observed in the morning at rural parks than urban parks. Rural parks had a lower percentage of 0–5 yrs old child visitors than urban parks, with no differences in the percentage of visitors from the other age groups. A greater (*p* < 0.03) percentage of visitors at rural parks was observed in sitting intensity activities and a greater (*p* < 0.003) percentage of visitors at urban parks was observed in moderate intensity activities. The overall MET intensity of the observed visits did not differ (*p* < 0.87) between urban and rural parks. When including population as a covariate, the visits per amenity was greater at urban parks.

As shown in Table 4, of all visitors observed, a greater percentage of those visitors were observed in picnic shelters at rural parks. A greater percentage of visitors were observed on sports fields and spectating sports events in urban parks. A greater percentage of adults (those visitors 19+ years) at urban parks were observed spectating others engaged in active play on playgrounds and in sports.

As shown in Table 5, there were significant (*p* < 0.001) age group differences by setting (rural, urban) interactions for visitation of some park areas. A greater (*p* < 0.05) percentage of rural park visitors of all age groups were observed at picnic shelters, but the setting difference was most marked for adolescents aged 13–18 years. Rural children aged 0–12 years were more likely to be observed engaged in sports than their urban counterparts. However, the opposite was observed for adolescents aged 13–18 years, as a greater proportion of adolescents at urban parks were observed engaged in sports than rural adolescents. A greater proportion of urban youth age 0–12 years were observed at a playground. A main effect of setting was observed for open space as a greater (*p* < 0.003) proportion of people in rural parks were observed in open space than in urban parks.

A greater (*p* < 0.03) proportion of people were observed on walking paths in rural parks and there was also a main effect of age (*p* < 0.001) as a lower proportion of 0–12 year old youth were observed on paths than 13–18 year old youth (*p* < 0.008) or people 19+ years (*p* < 0.001). The only significant effect for MET intensities at specific amenities was an age effect (*p* < 0.001) for playgrounds as the children age 0–12 years were more (*p* < 0.001) intensely active than the 19+ age group.

## 5. Discussion

The rural areas of America are extremely diverse in ethnicity of their peoples, geography, climate, terrain, forestation, prevalent occupations, educational attainment, traditions, customs, culture, attitudes, values, and many other factors [30]. Thus, to understand the influence of parks on the physical activity of those living in rural communities, it is necessary to study physical activity in a variety of rural areas. These same diversity issues are present when attempting to extend research results of the influence of built environment on sedentary and physical activity behaviors from urban areas to rural communities [31,32]. For example, Behavioral Risk Factor Surveillance System data revealed that rural–urban differences in the prevalence of meeting physical activity guidelines and the prevalence of physical inactivity were area dependent, with the greatest rural–urban differences occurring in the Southern U.S. [31]. However, there were only very slight rural–urban differences in the prevalence of sedentary behavior and meeting of physical activity guidelines in the Midwestern U.S. [31]. In agreement, Shores and West [33], examined 4 rural and 4 urban parks in the Southern U.S. (North Carolina (NC)) and found that urban park visits were more likely to include physical activity and rural park visitors more likely to be observed in sedentary behavior.

Given these possible regional differences in sedentary and physical activity behavior, the present study extends the research on park visitation, amenity use, and physical activity to residents of rural and urban communities in the Midwest U.S., specifically the Northern Plains. Northern North Dakota (ND) differs in ethnicity, geography, climate, terrain, forestation, prevalent occupations, and some customs, culture, attitudes, and values compared to the research gathered from the 4 urban and 4 rural parks in NC. The present study also extends previous research by observing 15 urban parks and 15 rural parks matched for acreage and amenities. However, despite the many regional differences, two main results in the present study are well aligned with those of the study conducted in NC [33] in that a greater percentage of rural park visitors were observed in sedentary (sitting-intensity) activities and a greater percentage of urban park visitors engaged in moderate-intensity physical activity.

In the present study, shelters were used by a greater percentage of rural park visitors of all age categories. Rural parks serve as community gathering places, helping to maintain the easy-going communal nature in a rural population [34]. Shelters are used for picnics and gatherings, and tend to support less physical activity than other amenities [35].

This difference in activity intensity between urban and rural park users may be due to cultural differences between the residents of rural and urban settings that influence how residents perceive and utilize their surrounding built environment differently for physically active purposes [11]. The 2014 Update of the Rural–Urban Chartbook [36] describes a greater likelihood of smoking, consuming alcohol, of being physically inactive during leisure time, and a greater incidence of obesity in rural communities. This pattern of health behaviors has led some to suggest that a “rural culture” health determinant is at play, and that environmental and cultural factors are affecting health behaviors [37]. As discussed above, there is wide diversity among individuals living in rural settings and in rural communities. Hence, though it is important to better understand the cultural issues that lead to reduced physical activity and other unhealthy behaviors in rural areas, these cultural issues will likely differ between communities. That said, cultural issues that may be impeding adoption and adherence to health behaviors, such as physical activity in North Dakota, include a health care workforce shortage, a need for more behavioral health professionals, affordability of health care and insurance, distances to parks and other physical activity outlets, a lack of community wellness and fitness facilities, fewer options for developing individualized social networks whose members provide social support of park visitation for physical activity, and fewer organized child care and youth activities [17,18].

However, there are also differences in results between the previous work in NC and the current work in ND. The rural parks in NC [33] were visited more frequently than the urban parks, whereas the rural parks in ND were visited less frequently, even when covarying for population. The rural community in NC had a population of almost 43,000, while the urban community in ND had a population of 65,534, and the rural communities had a mean population of 833 people. Perhaps rural ND is so sparse in population that visiting a park requires a greater travel distance than the rural community studied in NC. In addition, opposite of the parks in NC [33], the rural parks in ND were visited proportionally more in the morning.

The present study also extends the work of Shores and West [33] by studying the association of rural and urban park programming with visitation and physical activity [38]. Availability of park staff and programming promotes park use and physical activity [39], whereas a decrease in scheduled programs reduces park visitation [40]. Encouragingly, the number of days of sports and other programming, as measured, did not differ between rural and urban parks. Despite the similarities in days of sports programming, a greater percentage of visitors, especially adolescents, were observed on urban than rural park sports fields. This could be the result of urban adolescents having a greater population of youth to join in playing an organized or unorganized game or sport during summer vacation (Table 1); the easier access of urban youth to get to the park, not having to drive considerable distances from a farm into town to engage in an unorganized activity; greater sidewalk safety of those adolescents living in town who are willing to actively commute to the park (Table 1); and the greater physical active quality score of the urban parks (Table 1). Park access is associated with physical activity for adolescents in urban, but not rural areas [12]. A great percentage of park-based physical activity occurs on sports fields [41], and sports fields promote adolescent moderate-to-vigorous physical activity [42].

Few studies have objectively observed the physical activity levels at urban and rural parks [33]. While most adolescents were observed on sport fields, younger children were observed on playgrounds. Playground use also differed in rural and urban settings. Playgrounds were used by a greater percentage of children in urban parks than in rural parks, and children’s activity intensity at playgrounds is often of moderate intensity or greater [42]. Playgrounds are an especially important resource for promoting physical activity of younger children [43], and the greater use of urban playgrounds could also explain the greater activity intensity in urban parks.

Park visitation declines sharply from childhood to adolescence [44], as confirmed in the present study. However, it is important to understand what amenities or programming will increase adolescents park visitation, as access to safe parks is positively associated with physical activity, at least in urban adolescents, but not in rural adolescents [12]. The present study confirms previous research [44] that fewer adolescents than children are observed using playgrounds, but that there are more adolescents in shelters and sports courts; in addition, this study extends upon this area of research by showing that rural adolescents are more likely to be observed in shelters while urban adolescents are using sports fields. Adolescents have outgrown most playground equipment designed for younger youth, so another type of amenity, such as a sport field, is needed to promote physical activity. However, renovations of sports fields alone are ineffective for increasing teen park use [38], and sport fields and courts usually require a number of teens to play together in an organized fashion, and as described above, the travel distances and quality of sports facilities may act as barriers for such activities. Park programming aimed towards adolescents would increase adolescent activity. For example, greater park programming encourages adolescent girls to visit a park [38] and adding classes at a skate park increases teen visitation and activity [45]. The addition of skate parks or playground equipment specifically designed for adolescents would increase activity [40,46] for those youth who visit the park alone, or who prefer to not play sports.

This study is not without limitations. The rural parks had lower EAPRS quality, access, and sidewalk safety scores. However, only the EAPRS access score predicted park visitation. Thus, only one these three differences between rural and urban parks may have influenced the visitation results. The EAPRS “access” factor is scored from such attributes as the number, size, and/or proximity of entrances, parking lots, bike racks, lighting, and restrooms, as well as sidewalks, and roadways through parks. Given the low population density of the northern plains, we chose to study almost all of the parks in the small rural communities within 65 miles of the urban center. Thus, within this geographic region of the United States, there was no way to match on EAPRS quality, access, and sidewalk safety, as they are built to attribute differences between rural and urban community parks. Rural areas in the United States can differ in so many cultural and geographic attributes that may influence park visitation, amenity use, and physical activity intensity, so extension of the present results to other rural areas should be made with caution.

## 6. Conclusions

Rural parks in ND, matched with urban parks for size and amenities, promoted lower visitation and less physical activity. These results, along with those of Shores [33], demonstrate that findings from urban park studies should not be considered representative of their rural counterparts. Parks can be a valuable resource for physical activity, especially those parks in rural communities where there are few other options for being physically active. Therefore, it is important to identify the specific park characteristics that will promote park visitation and physical activity in rural areas.

## Figures and Tables

**Table 1:** Study parks’ area and amenities.

Rural														
8.7 ac	2.4 ac	21.4 ac	13.5 ac	2.1 ac	7.2 ac	4.9 ac	10.2 ac	8 ac	14.9 ac	21.3 ac	24.1 ac	12.8 ac	2.4 ac	6.7 ac
tennis	soccer	tennis	tennis	hockey	football	baseball	volleyball	baseball	play	tennis	play	baseball	tennis	tennis
football	b-ball	baseball	baseball	play	baseball	play	baseball	play	shelter	volley	shelter	play	b-ball	baseball
b-ball	hockey	b-ball	hockey		play	shelter	play	shelter	path	baseball			play	play
hockey	play	play	play		shelter		shelter			play			shelter	
play	shelter	shelter	shelter											
Urban														
17.5 ac	1.6 ac	10.0 ac	7.0 ac	4.0 ac	5.3 ac	6.0 ac	10.2 ac	7.9 ac	18.0 ac	20.0 ac	11.7 ac	5.0 ac	7.4 ac	18.8 ac
tennis	soccer	soccer	baseball	hockey	baseball	baseball	soccer	baseball	play	soccer	play	baseball	soccer	tennis
soccer	hockey	baseball	b-ball	play	hockey	play	football	b-ball	shelter	volley	shelter	b-ball	baseball	soccer
football	play	b-ball	play		play	shelter	b-ball	play	path	b-ball	path	play	hockey	baseball
baseball	shelter	hockey	shelter		shelter		hockey	shelter		play			play	b-ball
hockey		play					play			shelter				hockey
play							shelter							play
shelter														shelter
path														path

ac: acres; b-ball: basketball; play: play; path: walking/bicycling path; volley: volleyball.

**Table 2:** Park and neighborhood level characteristics of rural and urban settings.

	Rural	Urban	*p*
**Park level**			
Acreage	10.7 ± 1.8	10.0 ± 1.5	0.72
POST ^a^ score	43 ± 4	41 ± 4	0.52
EAPRS ^b^ score	589 ± 41	694 ± 64	0.17
Playsets score	173 ± 16	197 ± 12	0.24
Physical active quality score	0.72 ± 0.04	0.87 ± 0.02	0.006
Access score	17.8 ± 2.2	50.9 ± 5.7	<0.001
Sidewalk safety	30.9 ± 0.9	62.5 ± 3.7	<0.001
**Neighborhood level**			
Population ^c^	833 ± 141	1557 ± 728	0.007
Median household income	$53,831 ± 3535	$50,025 ± 5445	0.61
Walkscore	31 ± 4	49 ± 5	0.025

Mean ± SE; ^a^ POST = Public Open Space Tool; ^b^ EAPRS = Environmental Assessment of Public Recreation Spaces; ^c^ Population is the census population of the entire town for the rural settings and for the census block that the park resides in for the urban settings.

**Table 3:** Visitation patterns and activity intensity at rural and urban parks ^a^.

	Rural	Urban
Gender		
Females	44.5 (346)	46.1 (2295)
Males	55.5 (435)	53.9 (2410)
Day of week		
Weekday	82.4 (540)	81.9 (2496)
Weekend day	17.6 (241)	18.1 (2209)
Time of day		
1030 ^b^	51.7 (423)	30.6 (1468)
1430	19.1 (171)	28.0 (1647)
1730	29.2 (187)	41.1 (1590)
Age category		
0–5 yrs ^b^	6.0 (70)	14.9 (499)
6–12 yrs	34.1 (286)	31.6 (1146)
13–18 yrs	14.1 (88)	10.8 (888)
19+ yrs	45.8 (337)	42.7 (2172)
Activity intensity		
Sitting ^b^	39.9 (322)	27.4 (1717)
Standing	11.6 (95)	10.9 (637)
Moderate ^b^	34.0 (240)	48.0 (1828)
Vigorous	14.5 (124)	13.7 (523)
Overall METs	2.9 ± 0.2	2.7 ± 0.1
Visits per amenity ^b,c^	4.5 ± 1.9	22.6 ± 9.4

^a^ All values are % (N) of total visits, except overall METs and visits/amenity (data are mean ± SE). Percentages estimated using a generalized linear mixed model in which setting (rural or urban) was a fixed effect, and park pair was a random effect. ^b^ Values within row are significantly different, *p* < 0.05. ^c^ Covaried for population of the town for the rural settings and for the census block that the park resides in for the urban settings.

**Table 4:** Percentage of all visits observed at amenities in rural and urban parks ^a^.

	Rural % (N = 781)	Urban % (N = 4705)	*p*
Shelters	25.6	6.1	<0.001
Sport fields	16.1	22.7	<0.001
Playgrounds	27.5	26.8	0.66
Spectators	11.3	24.7	<0.001
Sport courts	4.9	3.9	0.22
Open space	4.1	1.9	<0.001
Treed area	5.0	3.2	0.01
Walking path	5.3	4.2	0.20
Gardens	0.0	2.2	0.97

^a^ Percentages estimated using a generalized linear mixed model in which setting (rural or urban) was a fixed effect, and park pair was a random effect.

**Table 5:** Percentage of visitors engaged in activities and MET intensity of those activities by age group at rural or urban parks.

	Rural		Urban	
	% of Visitors (*N* = 781)	METs	% of Visitors (*N* = 4705)	METs
**Shelters** ^a^				
0–12 yrs	11.0 *	1.7 ± 0.2	3.5 *	2.2 ± 0.2
13–18 yrs	34.1 *	1.7 ± 0.3	1.2 *	1.5 ± 0.3
19+ yrs	38.9 *	1.7 ± 0.2	10.1 *	1.5 ± 0.1
**Sport fields** ^a^				
0–12 yrs	25.0 *	2.2 ± 0.4	14.5 *	2.8 ± 0.1
13–18 yrs	8.0 *	3.6 ± 2.4	69.6 *	3.1 ± 0.6
19+ yrs	8.9	2.0 ± 0.1	9.7	2.8 ± 0.2
**Playgrounds** ^a,d^				
0–12 yrs	39.9 *	3.8 ± 0.3	51.0 *	3.5 ± 0.2
13–18 yrs	13.6	3.1 ± 0.9	9.2	3.0 ± 0.4
19+ yrs	18.1	2.5 ± 0.4	15.6	2.2 ± 0.1
**Open space** ^b^				
0–12 yrs	5.1	3.0 ± 0.3	1.9	3.3 ± 0.4
13–18 yrs	3.4	4.5 ± 1.5	2.3	3.3 ± 0.9
19+ yrs	3.6	2.4 ± 0.3	1.7	2.9 ± 0.6
**Walking path** ^b,c^				
0–12 yrs	2.8	3.6 ± 0.6	2.0	3.2 ± 0.3
13–18 yrs	8.0	4.5 ± 1.5	3.6	4.1 ± 0.7
19+ yrs	7.1	3.9 ± 0.7	6.2	3.6 ± 0.3

MET data are mean ± SE. Percentages estimated using a generalized linear mixed model in which setting (rural or urban), age and age × setting interaction were fixed effects, and park pair was a random effect. * Values within row, *p* < 0.05. ^a^ Age by setting interaction for % of visitors, *p* < 0.05. ^b^ Main effect of setting for % of visitors, *p* < 0.05. ^c^ Main effect of age, *p* < 0.05; a lower proportion of 0–12-year-old youths were observed on paths than 13–18-year-old youths or people 19+ years. ^d^ Main effect of age, *p* < 0.05; children aged 0–12 years were more intensely active than the 19+ age group.
